# Tumor Volume Kinetic Analyses Might Explain Excellent Prognoses in Young Patients with Papillary Thyroid Carcinoma

**DOI:** 10.1155/2020/4652767

**Published:** 2020-07-18

**Authors:** Toshihiko Kasahara, Akira Miyauchi, Yasuhiro Ito, Takumi Kudo, Hiroo Masuoka, Takuya Higashiyama, Mitsuru Ito, Minoru Kihara, Akihiro Miya

**Affiliations:** ^1^Department of Internal Medicine, Kuma Hospital, 8-2-35 Shimoyamate-dori, Chuo-ku, Kobe 650-0011, Japan; ^2^Department of Surgery, Kuma Hospital, 8-2-35 Shimoyamate-dori, Chuo-ku, Kobe 650-0011, Japan

## Abstract

**Introduction:**

Young patients with papillary thyroid carcinoma (PTC) generally have excellent prognoses despite their often-advanced disease status. The reasons for this excellent prognosis are poorly understood.

**Objective:**

To investigate the natural history of PTC in young patients, we compared the observed tumor volume-doubling rate (TV-DR) with the hypothetical tumor volume-doubling rate (hTV-DR) before presentation in young PTC patients. DR is an inverse of the doubling time and indicates the number of doublings that occur in a unit of time. A negative value indicates the number of times the volume is reduced by half per unit time.

**Methods:**

We enrolled 20 patients with the following characteristics: age ≤19 years, diagnosed with PTC according to the cytology results between 2013 and 2018 and followed-up with periodical ultrasound examinations for ≥3 months before surgery for various reasons. Seventeen patients later underwent surgery confirming the diagnosis. We calculated TV-DRs using serial measurements of tumor diameters after presentation and hTV-DRs using tumor diameters and patients' age at presentation, assuming that a single cancer cell was present at the patient's birth and that the tumor grew at a constant rate. These values indicate the lowest growth rates necessary for a single cancer cell to achieve the full tumor size at presentation.

**Results:**

Thirteen patients had positive TV-DRs (/year) ranging from 0.09 to 1.89, indicating tumor growth, and the remaining seven patients had negative values (−0.08 to −1.21), indicating regression. The median TV-DR was 0.29. The hTV-DRs (1.48–2.66, median 1.71) were significantly larger than the TV-DRs (*p* < 0.001), indicating much faster growth before presentation.

**Conclusions:**

These data suggest that deceleration of tumor growth had already occurred at presentation in the majority of the cases. This might explain why disease-specific survival is excellent despite frequent findings of advanced disease in young patients with PTC.

## 1. Introduction

Papillary thyroid carcinoma (PTC) is the most common type of thyroid differentiated cancer. The staging system most often used for thyroid cancers is the 8^th^ edition of the TNM classification of the American Joint Committee on Cancer (AJCC/TNM classification). All patients younger than 55 years at diagnosis with PTC are considered to have stage I cancer if they have no distant metastasis and stage II cancer if they have distant metastasis regardless of any T or N [1]. This classification is based on the excellent prognosis of patients with PTC younger than 55 years. Especially in younger age groups, i.e., children or adolescents with PTC, disease-specific survival is extremely good, with a 20-year survival rate of almost 100%, despite a particularly high frequency of lymph node metastasis and distant metastasis [2–4].

Collins et al. studied changes in tumor sizes of pulmonary metastases over time and proposed the concept that human tumors grow exponentially [5]. Moreover, Miyauchi et al. demonstrated that serum thyroglobulin values among patients with PTC after total thyroidectomy measured under a thyrotropin-suppressed condition also changed exponentially over time and reported that the thyroglobulin-doubling time was a strong prognostic factor [6]. Furthermore, Sabra et al. reported that thyroglobulin-doubling time correlated with tumor volume-doubling time in patients with pulmonary metastases of PTC [7]. Use of doubling time based on the concept of exponential tumor growth has been accepted for not only metastatic tumors but also for primary tumors [8–12]. Thus, doubling time is a well-validated way to evaluate and express tumor volume kinetics over time. However, it has two major limitations: first, if some of the tumors show a decrease in tumor volume, their doubling times are given in negative values, which creates a discontinuity problem from positive doubling time values. Second, the magnitude of doubling time values is inversely proportional to the growth rate. The inverse of doubling time (i.e., 1/doubling time) resolves these limitations. Miyauchi et al. proposed calling this index “doubling rate (DR)”, as it indicates the number of times that doubling occurs in a unit of time [12]. Negative DR values indicate the number of times the volume is reduced by half per unit time.

PTCs in young patients have generally excellent prognoses, despite the extended disease status; however, little is known about the reasons for their good prognosis. In Japan, radioactive iodine treatment for children and adolescents with PTC has been performed only for patients with distant metastasis, and thyroid ablation with radioactive iodine is rarely performed for patients aged <20 years. However, even in patients with PTC recurrence in the regional lymph nodes who did not receive radioactive iodine treatment postoperatively, the prognoses were good. We previously reported that postoperative thyroglobulin-doubling rates in PTC patients aged ≤18 years were significantly smaller or negative than their hypothetical tumor volume-doubling rates (hTV-DRs) before presentation (described below), irrespective of radioactive iodine administration, indicating that the growth of PTCs in children and adolescents spontaneously slowed or even regressed postoperatively [13].

Miyauchi et al. calculated tumor volume-doubling rates (TV-DRs) for adult papillary thyroid microcarcinomas (PTMCs) during active surveillance and reported that tumor growth, stabilization, and regression occurred in 25%, 57%, and 17% of their cases, respectively [12], and that hTV-DRs before presentation were significantly larger than TV-DRs observed during the active surveillance; the authors speculated that the tumors grew much faster before presentation.

When tumor dormancy occurs in some PTCs, rather than consistently following the classical exponential tumor growth model, exponential growth may be followed by a model of slow or regressive growth at some point in the patient's life. We hypothesized that tumor dormancy could be applied to young patients with PTC. This study aimed to reveal the changes in tumor growth rate by comparing TV-DR after presentation and hTV-DR before presentation in young patients with PTC.

## 2. Materials and Methods

Forty-five patients younger than 19 years were diagnosed as having PTC on the basis of cytology results between July 2013 and June 2018. Among them, we enrolled 20 patients who were followed with periodical ultrasound examinations for ≥3 months before surgery for various reasons, mostly parental hesitation to approve surgery. Of the 20 patients, 16 were incidentally detected by health check or imaging studies for some other purpose, and four were referred for a palpable mass. Chest computed tomography scan was performed in 19 cases with negative results, while one patient did not receive this examination.

Ultrasound measurements of tumor size were performed by well-trained ultrasonographers who specialized in thyroid examinations, and two thyroidologists confirmed the measurements on images of all patients.

We calculated their TV-DRs based on two or serial measurements of the tumor with ultrasound. Tumor volumes were calculated using the maximum diameter (*D*1), the diameter perpendicular to the maximum diameter (*D*2), and the tumor depth (*D*3). Tumor volume (*V*) was calculated using the volume equation (*π*/6 × *D*1 × *D*2 × *D*3) [7]. Time (*T*) was the interval from diagnosis to the last measurement of tumor size. The tumor volume-doubling time was calculated using the least squares method as previously reported [14]:

(1) $$\begin{array}{c} \text{Tumor volume}‐\text{doubling time}=\frac{\left(\log\;2\right)}{\alpha },\\ \alpha =\frac{\left(\left.n\sum_{k=1}^{n}T_{k}\times \log\left(V_{k}\right)-\sum_{k=1}^{n}T_{k}\times \sum_{k=1}^{n}\log\left(V_{k}\right)\right)\right.}{{\left(\sum_{k=1}^{n}T_{k}^{2}-\left(\sum_{k=1}^{n}T_{k}\right)\right)}^{2}}. \end{array}$$

Then, TV-DR was the inverse of the tumor volume-doubling time.

We calculated the hTV-DR before presentation in individual patients, using age and tumor size at presentation, assuming that a single cancer cell measuring 0.01 mm in diameter was present at birth and grew at a constant rate [12, 13]. The actual tumor growth rate should have been more rapid than the hypothetical DR value because the origin of the cancer should occur after birth.

To simplify the calculation of doubling times and DR values, we created a “Doubling Time, Doubling Rate, and Progression Calculator,” which can be downloaded from our homepage (http://www.kuma-h.or.jp/english/).

## 3. Statistical Analysis

We used the Wilcoxon signed-rank test to evaluate the difference between TV-DRs and hTV-DRs. In the analysis regarding the comparison of TV-DRs according to the length of observation, we used the Mann–Whitney *U* test. All statistical analyses were performed using StatFlex software (version 6.0; YUMIT, Osaka, Japan). All statistical tests were two-sided, with the level of significance set at *p* value < 0.05.

## 4. Results

The patients consisted of 17 female and three male patients aged 12–19 years (median, 18 years) (Tables 1 and 2). Thyroidectomy was performed within 7 months (median, 4 months; range, 3–7 months) in 15 of 20 patients. Of the remaining 5 patients who refused surgery at diagnosis, 2 underwent surgery later: 1 after 19 months with tumor enlargement and 1 after 40 months with the tumor size unchanged. None of the tumor components (i.e., solid, mixed, or cystic) changed during the course. The 17 surgical cases were all pathologically confirmed as PTC, 15 of which were classical PTC, 1 was cribriform variant, and 1 was follicular variant PTC. Among the surgical cases, one was pT1a, three were pT1b, three were pT2, and 10 were pT3b, while four were cN0, six were pN1a, and seven were pN1b. Three patients were retained for watchful observation with a median follow-up of 51 months (range, 30–60 months). These three patients had T1aN0M0 disease at diagnosis.

**Figure 1:**
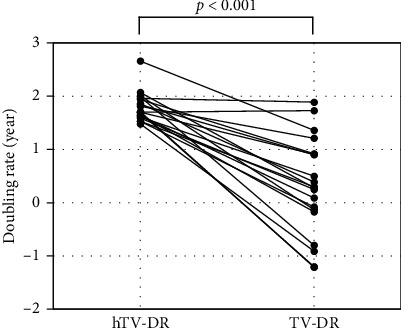
Comparison of TV-DRs and hTV-DRs. The difference in TV-DRs was calculated using the Wilcoxon signed-rank test. In the 20 patients, the TV-DRs after diagnosis were smaller than the hTV-DRs, and seven patients had negative values, suggesting slowing or regression of the tumors (*p* < 0.001). TV-DR: tumor volume-doubling rate after presentation; hTV-DR: hypothetical tumor volume-doubling rate before presentation.

**Figure 2:**
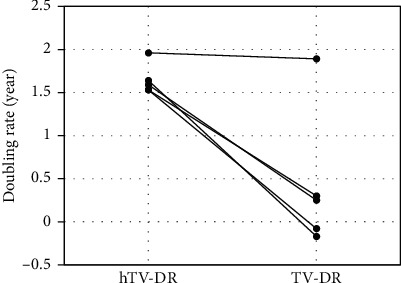
Comparison of TV-DRs and hTV-DRs in five patients who refused surgery at diagnosis. Five patients refused surgery at diagnosis; of these, two underwent delayed surgery, and the remaining three were under active surveillance. Although there was no significant difference because of the small number of cases, the hypothetical TV-DRs (median 1.59; range, 1.53–1.96) were larger than the TV-DRs in all cases (ns), indicating faster growth before presentation. One patient underwent surgery 19 months after diagnosis because of tumor growth. Even in this progressive case, hTV-DR (1.96) was larger than TV-DR (1.89) under observation. TV-DR: tumor volume-doubling rate after presentation; hTV-DR: hypothetical tumor volume-doubling rate before presentation.

Thirteen patients had positive TV-DRs (/year) ranging from 0.09 to 1.89 indicating slow to rather rapid growth, and the remaining seven had negative values (−0.08 to –1.21), indicating even regression. The median TV-DR was 0.29. While hTV-DRs ranged from 1.48 to 2.66 (median, 1.71), which was significantly larger than the TV-DR values (*p* < 0.001, Figure 1), indicating much faster growth before presentation. Five patients who refused surgery at diagnosis had an observation period of 19–60 months (median, 40 months) with a median TV-DR of 0.25 (individual TV-DRs were −0.17, −0.08, 0.25, 0.3, and 1.89). Although there was no significant difference because of the small number of cases, the hTV-DRs (median 1.59; range, 1.53–1.96) were larger than TV-DRs in all five cases (ns, Figure 2).

As the short observation period may have caused imprecision in the DR calculation, we compared TV-DRs in cases with observation periods of 3–6 months (14 cases) and >6 months (6 cases). The TV-DRs of 14 patients whose observation period was 6 months or less ranged from −1.21 to 1.73 (median, 0.45), and those of 6 patients whose observation period was >6 months ranged from −0.91 to 1.89 (median, 0.085). We found no significant difference in TV-DRs according to the length of observation.

## 5. Discussion

In this study, we compared the hypothetical tumor growth rate, calculated as hTV-DR before presentation, with the observed tumor growth rate, calculated as TV-DR in pediatric/adolescent PTCs. Although hTV-DR before presentation indicated rather rapid tumor growth before presentation, PTCs in young patients had already slowed or even decreased in size at the time of presentation.

The hTV-DRs were calculated using the tumor diameters and the patient's age at diagnosis, assuming that a single cancer cell was present at the patient's birth and grew at a constant rate. Tumors may have developed later than birth or may have been growing at variable rates before diagnosis. That is, if there was an initial period of no tumor and no growth, or a period of slow growth, then a more rapid growth period than the hTV-DR value was required for subsequent tumor growth to reach the presentation size. It means that the actual TV-DR before presentation was larger than the hTV-DR value, and based on the results of this study where the TV-DR value after presentation was smaller than the hTV-DR value, it indicates that tumor growth has already slowed. If there was an initial period of more rapid growth, the tumor would grow larger than it was at diagnosis in the absence of a subsequent period of slower growth. In other words, this also means that growth had already slowed down before presentation.

Contrary to high hTV-DRs, TV-DRs after presentation were clearly lower. The classic tumor growth model shows exponential growth; however, there are reports of breast cancers, prostate cancers, and thyroid cancers, for which the tumors have a prognosis that varies with age at diagnosis [3, 9, 11, 12, 15, 16]. Our data suggest that even in young patients with advanced PTC, tumor dormancy might be observed at diagnosis in some cases. Hay et al. reported that the tumor size at diagnosis was greater in children than in adults [17]. Mazzaferri and Kloos reported that PTC patients younger than 19 years had a high incidence of local and distant recurrences, whereas their disease-specific survival was good [3]. Papac reported that a tumor could naturally regress [18]. Moreover, Miyauchi et al. reported that tumor regression was observed in 17% of cases, based on a 10-year-follow-up data of adult PTMCs [8]. In addition, Miyauchi et al. reported that the thyroglobulin-doubling time was negative in 17% of cases after total thyroidectomy in 426 adults with PTC [19]. Negative thyroglobulin-doubling time indicates tumor shrinkage. Furthermore, we reported that the growth of the PTCs in children and adolescents spontaneously slowed down or even regressed postoperatively regardless of the administration of radioactive iodine [13]. In the present study, 35% of PTCs in young patients exhibited negative TV-DRs, indicating shrinkage after diagnosis, despite suggesting initial rapid growth. Therefore, although PTC in young patients initially grows rapidly, the rate of subsequent tumor dormancy appears to be greater than that reported in adult patients. That seems to partially explain why young PTC patients have a favorable disease-specific survival.

This study has some limitations. In this study, the observation period of patients undergoing surgery was short, and the majority of patients had only two serial measurements, which could have led to imprecise calculations of DR; however, TV-DR has been reported to correlate with prognostic factors and disease-free survival in breast cancer at short observation periods [20]. Patients with an observation period of ≥3 months were enrolled to improve evaluation accuracy. Ultrasonographic examinations were performed by examiners who were well trained in neck ultrasonography. Variations in measured values of tumor diameter such as interobserver and intraobserver variations were resolved using confirmation by two thyroidologists and analysis using the least squares method. Moreover, the number of cases was small (20 cases) because a large number of patients underwent surgery within 2 months after diagnosis. However, these cases were registered from among 2756 children who visited a high-volume hospital in the 5-year study period.

## 6. Conclusion

In this comparative study of DRs, the median TV-DR was 0.29, suggesting slow growth of the tumor, while seven patients showed negative values. The median hTV-DR before presentation was 1.71, suggesting more rapid growth before presentation than that after presentation. These data suggest that deceleration of tumor growth had already occurred at presentation in the majority of young patients. This might be a clue to explain why disease-specific survival is excellent despite the frequent findings of advanced disease in pediatric/adolescent patients with PTC.

## Figures and Tables

**Table 1:** Clinical features of the 20 pediatric or adolescent patients with papillary thyroid carcinoma.

Clinical and biochemical features	
Age (years)	18 (12–19)
Male/female	3/17
Tumor size (mm)	16.5 (7–44)
cN0 or pN0/pN1a/pN1b (cases)	7/6/7
MX/M0/M1 (cases)	1/19/0
Follow-up period before surgery (months)	4.5 (3–60)
Cases underwent surgery	17
3 months after diagnosis	7
4 months after diagnosis	3
5 months after diagnosis	3
6 months after diagnosis	1
7 months after diagnosis	1
19 months after diagnosis	1
40 months after diagnosis	1
Cases followed without surgery	3
30 months after diagnosis	1
51 months after diagnosis	1
60 months after diagnosis	1
TV-DR (/year)	0.29 (−1.21–1.89)
hTV-DR (/year)	1.71 (1.48–2.66)

Values indicate median (ranges) and number of cases. TV-DR: tumor volume-doubling rate; hTV-DR: hypothetical tumor volume-doubling rate before presentation.

**Table 2:** Clinical presentation and measurements of 20 cases followed for more than 3 months.

Case	Sex	Age at diagnosis (years)	Initial measurements of the tumor (mm)			Final measurements of the tumor (mm)			Follow-up period (months)	Number of measurements	TV-DR (/year)	hTV-DR (/year)
			*D*1	*D*2	*D*3	*D*1	*D*2	*D*3				
1	F	19	32	25	24	33	22	19	4	2	−1.21	1.71
2	M	19	26	16	16	25	16	13	3	2	−1.20	1.69
3	F	19	9	6	9	8	6	7	7	2	−0.91	1.48
4	M	16	24	20	17	26	17	16	3	2	−0.80	1.99
5	F	19	21	15	13	20	15	9	40	6	−0.17	1.64
6	F	15	16	12	10	15	14	12	6	4	−0.13	2.01
7	F	18	7	6	6	6	6	5	30	6	−0.08	1.53
8	F	18	12	9	8	14	8	8	4	3	0.09	1.64
9	F	18	10	9	7	12	10	10	51	10	0.25	1.59
10	F	15	18	17	14	20	18	13	5	2	0.28	2.07
11	F	18	9	8	6	15	10	8	60	7	0.30	1.53
12	F	17	27	20	17	29	20	17	3	2	0.39	1.86
13	F	19	16	10	11	17	10	12	5	2	0.50	1.61
14	F	15	10	7	6	13	7	6	5	2	0.90	1.84
15	F	16	7	5	7	7	6	7	3	2	0.91	1.70
16	F	16	31	17	16	29	19	18	3	2	0.92	1.94
17	M	19	44	38	28	43	42	34	3	2	1.21	1.81
18	F	12	29	24	15	35	24	16	3	2	1.36	2.66
19	F	18	17	12	10	19	14	12	4	2	1.73	1.70
20	F	13	8	4	4	15	12	7	19	4	1.89	1.96

*D*1: maximum tumor diameter, *D*2: diameter perpendicular to the maximum tumor diameter, and *D*3: depth (mm). TV-DR: tumor volume-doubling rate; hTV-DR: hypothetical tumor volume-doubling rate before presentation. Cases 7, 9, and 11 were under active surveillance at the end of the study period. Cases 5 and 20 underwent thyroidectomy after 19 and 40 months of follow-up, respectively.

## Data Availability

Data cannot be shared publicly because of privacy. Data are available from the Ethical Committee at Kuma Hospital (contact via authors) for researchers who meet the criteria for access to confidential data.
